# Anti-Inflammatory Effect of Sparstolonin B through Inhibiting Expression of NF-κB and STAT-1

**DOI:** 10.3390/ijms231810213

**Published:** 2022-09-06

**Authors:** Nayeon Kim, Chaeyeong Kim, Soo Ho Ryu, Go Oun Kim, Jong-Sup Bae

**Affiliations:** College of Pharmacy, Research Institute of Pharmaceutical Sciences, Kyungpook National University, Daegu 41566, Korea

**Keywords:** Sparstolonin B, endothelium, iNOS, p-STAT-1

## Abstract

Sparstolonin B (SsnB), which is found in *Sparganium stoloniferum,* prevents the synthesis of inflammatory mediators and is related to functional pathways of survival. In this study, we assessed the possible protective functions of SsnB on lipopolysaccharide (LPS)-induced inflammatory responses. We determined the functions of SsnB on controlling heme oxygenase (HO)-1, cyclooxygenase (COX-)2, and inducible nitric oxide synthase (iNOS) in LPS-activated human umbilical vein endothelial cells (HUVECs). Furthermore, the distinct function of SsnB on the expression of iNOS and well-known pro-inflammatory mediators, such as tumor necrosis factor (TNF)-α and interleukin (IL)-1β, were assessed in the pulmonary histological status of LPS-injected mice. SsnB upregulated the HO-1 production, inhibited luciferase-NF-κB interaction, and lowered COX-2/PGE2 and iNOS/NO, which lead to the reduction of STAT-1 phosphorylation. Moreover, SsnB enhanced the nuclear translocation of Nrf2, elevated the binding activity between Nrf2 and antioxidant response elements (AREs), and weakened IL-1β expression on LPS-treated HUVECs. SsnB-suppressed iNOS/NO synthesis was restored by the process of the RNAi inhibition of HO-1. In experiment with an LPS-injected animal model, SsnB remarkably decreased the iNOS expression in the pulmonary biostructure and TNF-α level in the bronchoalveolar lavage fluid (BALF). Therefore, these results demonstrate that SsnB is responsible for inflammation ameliorative activity by controlling iNOS through inhibition of both NF-κB expression and p-STAT-1. Therefore, SsnB could be a candidate for promoting novel clinical substances to remedy pathologic inflammation.

## 1. Introduction 

Preventing against inflammation-inducing damage and accumulation of ROS, heme oxygenase-1 (HO-1) has a biochemical role in the landscape of lethal illness, such as acute autoimmune response, lung conditions, and malignant tumors [1,2]. HO-1 inhibits the synthesis of proinflammatory cytokines and factors, such as tumor necrosis factor (TNF)-α, and interleukin (IL)-1β and IL-6 [2]. Several previous studies have proven that HO-1 has a suggestive impact on protecting cecal ligation and perforation-induce sepsis in mice from acute septic inflammation [2,3]. HO-1 mediated regulation showed a significant clinical effect on a number of vascular illnesses related to inflammation [2]. The overexpression of the HO-1 gene is co-regulated with nuclear factor erythrocyte 2-related factor 2 (Nrf2), which makes complex with antioxidant response elements (AREs) that are coded on the sequence of promoters where antioxidant enzyme genes exist. The Kelch-like ECH-associated protein 1 (Keap1)-Nrf2-ARE pathway is principal in the adjustment of oxidative stress and homeostasis of the reverse inflammatory reaction by not only regulating antioxidant genes, which mediate transcription process for conservation of cytoplasmic constancy, but detoxification genes for operating and degrading cancer-causing agents and toxic elements before injury [4]. Nrf2 acts as a major factor in the modulation of cytoprotective action in response to extracellular conditions [4]. In normal biochemical environments, Nrf2 normally resides in the cytosol because of its actin-binding negative regulator protein Keap1, a zinc metalloprotein located nearby the cell membrane [4]. On the other hand, in the condition of oxidative stress, the Nrf2-Keap1 interplay is triggered in a concentration-dependent manner, and the isolated and freshly produced Nrf2 migrates into the nucleus, where it organizes heterodimers with one of the musculoaponeurotic fibrosarcoma oncogene homolog proteins [4]. Then, Nrf2-Maf binding structure facilitates the ARE-mediated gene translation and protein expression of a chain of cellular defensive and antioxidative proteins. Furthermore, the Nrf2/Maf/ARE multistructure contributes inflammation-suppressing, antioxidant, detoxicative, autophagy-related, and proteasomal functions in physiological phenomena [4]. The synthesis of antioxidative proteins is activated to manage a variety of stresses, such as oxidative damage [5]. Therefore, the Nrf2-ARE pathway is recognized as a key target for the clinical approach of inflammation-mediated disorders [5]. Lung functional disorder, which is characterized by proliferative edema, blood-hypoxia, and neutrophil extracellular trapped inside the lung, is a lethal respiratory failure caused by inflammation. It is widely established that lipopolysaccharide (LPS) acts as main pathogenic factor of lung disorders [6]. LPS promotes the expressions of pro-inflammatory mediators through activation of transcriptional proteins. These inflammation-mediated molecules upregulate not only long-term inflammation but also cause the development of inflammatory disorders, s vascular dysfunction, asthma, chronic obstructive pulmonary disease (COPD), and cystic fibrosis [6].

**Figure 1:**
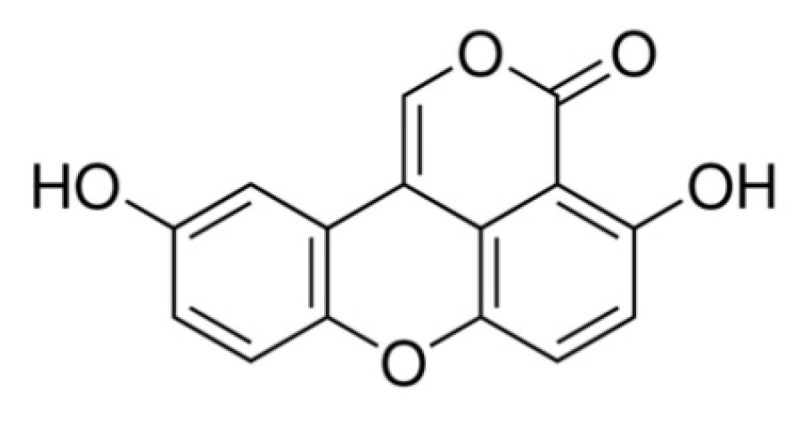
Chemical structure of Sparstolonin *B* (SsnB).

Sparstolonin *B* (SsnB, Figure 1) is found in the Chinese herb, *Sparganium stoloniferum* and effectively suppresses the expressions of IL-1, IL-6, and TNF-α in response to LPS stimulation [7]. Moreover, in a dose-dependent way, SsnB represses NF-κB activity regulated by toll-like receptor (TLR)4, swarms myeloid differentiation primary response gene 88 (MyD88) around TLR4, and reasonably diminishes LPS-caused inflammatory reaction in animal models [8,9]. As a selective TLR antagonist, SsnB showed significant pharmacological activities such as anti-tumour, anti-obesity, and anti-inflammatory effects in various inflammatory disorders [7,9,10,11,12,13,14]. Likewise, SsnB improved mouse survival when SsnB was injected before and after LPS treatment. SsnB also plays a protective role in mouse models with LPS-induced lung disease [8]. Additionally, SsnB blocks inflammatory reactions induced by LPS in 3T3-L1 adipocytes [13] and human umbilical vein endothelial cells (HUVECs) [14]. Still, how the activation network of SsnB suppresses HO-1 and other inflammatory mediators, such as TNF-α, IL-1β, and NO in HUVECs in vitro or histologic structure of LPS-injected mice in vivo remains unidentified. For this reason, the point of this study was to prove and define the role of SsnB on the triggering of HO-1 signal transduction and inhibition of inflammatory cytokines. Furthermore, we discovered how the prominent subprocess of SsnB works as a potential substance targeting inflammatory pathology.

## 2. Results

### 2.1. Inhibitory Effect of SsnB on the Expressions of iNOS and COX-2 by LPS

**Figure 2:**
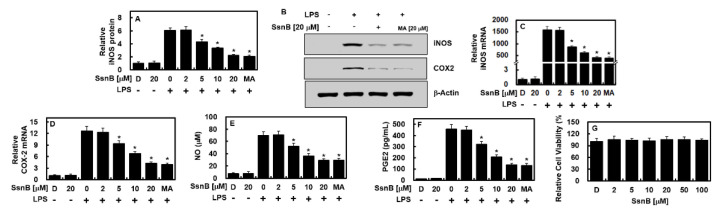
SsnB inhibited the expression levels of COX-2 and iNOS by LPS in HUVECs. After LPS stimulation at 1 μg/mL for 6 h, the HUVECs were activated with SsnB or MA (20 μM) for 6 h, and the levels of iNOS protein (**A**,**B**), COX-2 protein (**B**), iNOS mRNA (**C**), COX-2 mRNA (**D**), NO (**E**), and PGE2 (**F**) were analyzed. The results represent the mean ± standard deviation (SD) values. (**G**) The cellular viability of SsnB was measured using the MTT assay. D = 0.2% DMSO (vehicle control). * *p* < 0.05 vs. LPS.

The expressions of two recognized proinflammatory mediators, iNOS and COX-2, were estimated to investigate the activity of SsnB in the process of inflammation-related gene expressions. In this study, maslinic acid (MA) was used as a positive control [15]. After 6 hours of LPS treatment, the HUVECs were conditioned with different doses of SsnB or MA (20 μM) for 6 h. The data of the qPCR, ELISA, and immunoblot assays showed that the expressions of iNOS and COX-2 by LPS were downregulated along with the treatment of SsnB dose-dependently or MA at 20 μM (Figure 2A–D). To verify this result, we detected the expressions of their correlated molecules (PGE2 and NO), and it turned out that they were decreased after SsnB or MA administration (Figure 2E,F). In addition, the possible cytotoxicity of SsnB on HUVECs was determined by performing the MTT assay. As described in Figure 2G, there was no cell viability variation observed in HUVECs up to 100 μM of SsnB. These results indicated that SsnB effectively prevented LPS-induced NO synthesis by inhibiting iNOS production.

### 2.2. Inhibitiroy Effect of SsnB on the Activity of NF-κB and STAT-1 and the Expression of HO-1 by LPS

**Figure 3:**
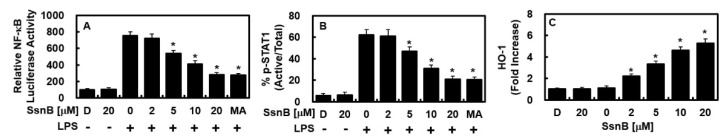
SsnB inhibited the activities of NF-κB and STAT-1 and enhanced HO-1 protein expression levels. HUVECs were treated with SsnB or MA (20 μM) for 6 h after LPS stimulation at 1 μg/mL for 6 h. (**A**) The activity of NF-κB was measured using NF-κB luciferase reporter system. (**B**) Phosphorylation of STAT1 (p-STAT1) by LPS was determined by ELISA. (**C**) HO-1 levels were determined by ELISA. The results represent the mean ± SD. D = 0.2% DMSO (vehicle control). * *p* < 0.05 vs. LPS.

Because NF-κB plays a major role in inflammation-related gene expression processes, the impact of SsnB on NF-κB regulation was investigated. As shown in Figure 3A, SsnB or MA repressed NF-κB luciferase reporter response in concentration-dependent manner. It had been reported that the JAK/STAT signaling cascade acts as a critical regulatory mechanism in the synthesis of iNOS and COX2 in an LPS-treated environment [16,17]. Therefore, SsnB not only suppressed STAT-1 phosphorylation and its product (Figure 3B) but considerably increased the level of HO-1 (Figure 3C).

### 2.3. Inhibitory Effect of SsnB on the Nuclear Transport Activity of Nrf2, Are Reporter, and Anti-Inflammatory Activity

**Figure 4:**
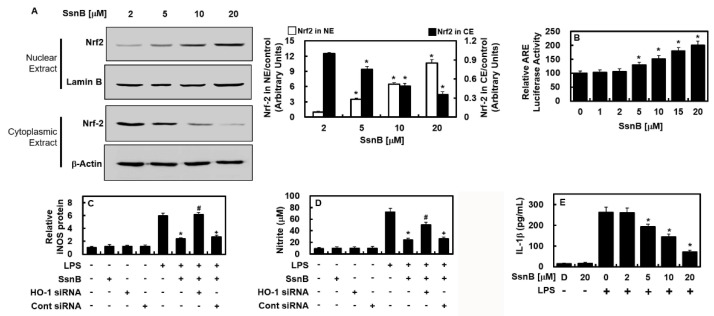
SsnB mediated the nuclear transport of Nrf2 and exerted anti-inflammatory functions. (**A**) Cells were incubated with SsnB for 6 h and then, cytosolic extract (CE) and nuclear extract (NE) were prepared. Western blotting (**left**) or densitometric intensity (**right**) was analyzed. (**B**) Relative ARE luciferase was analyzed after cells were transfected with ARE. Relative iNOS level (**C**) or level of NO (**D**) was measured after cells were suppressed with HO-1. (**E**) HUVECs were treated with SsnB for 6 h after LPS stimulation at 1 μg/mL for 6 h and then the levels of IL-1β were determined using ELISA kit. The results represent the mean ± SD. D = 0.2% DMSO (vehicle control). * *p* < 0.05 vs. LPS, # *p* < 0.05 vs. LPS + SsnB, or *p* < 0.05 vs. LPS + SsnB + HO-1 siRNA.

Noting that the expression of antioxidative proteins, such as HO-1, depends on Nrf2, we next tested the effects of SsnB on the nuclear localization of Nrf2 and the expressions of ARE. SsnB stimulated the nuclear transmigration of Nrf2 and upregulated ARE luciferase reporter response (Figure 4A,B). To verify whether the SsnB repression of iNOS expression was caused by the HO-1 production, the effect of HO-1 was repressed by employing siRNA. The suppression of HO-1 reacted in the recovery of iNOS and NO expressions similiarly to those in the cells without SsnB treatment (Figure 4C,D), which indicates SsnB improved HO-1 expression via repression of the iNOS level. The inflammation-alleviating function of SsnB was also illustrated by the downregulation of IL-1β expression in the LPS-treated HUVECs (Figure 4E).

### 2.4. Suppressive Effect of SsnB on LPS-Mediated TNF-α and iNOS Protein Levels In Vivo

**Figure 5:**
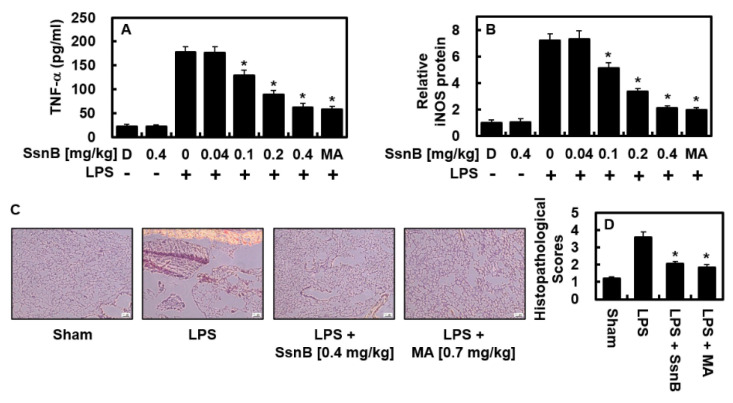
SsnB inhibited LPS-induced TNF-α and iNOS levels and mitigated lung injury in mice. Mice were injected with SsnB (0.04–0.4 mg/kg) or MA (0.7 mg/kg) intravenously 6 h after LPS (15 mg/kg) injection intraperitoneally (*n* = 5). Then, 24 hours after LPS injection, lung tissues or BALF was prepared, and the protein levels of TNF-α (**A**) and iNOS (**B**) were determined. The results represent the mean ± SD. D = 0.2% DMSO (vehicle control). (**C**) H&E staining of lung tissues from each group (*n* = 5). Scale bar = 160 μm. (**D**) Histopathological scores for each group were shown. * *p* < 0.05 vs. LPS.

Next, we investigated the anti-inflammatory functions of SsnB in vivo. As described in Figure 5A, the upregulated production of TNF-α by LPS was considerably decreased in SsnB- or MA (0.7 mg/kg)-treated BALF. An expected blood quantity of 72 mL/kg in mice [18,19] and the body weight of mice used in this study (27 g) were noted, and the average circulating blood volume was measured to be 2 mL. For this reason, a treatment of 0.04, 0.1, 0.2, or 0.4 mg/kg of SsnB was converted to calculated concentration of up to 2, 5, 10, or 20 μM, each, in the peripheral fluid. With the same pharmacological logic, MA at 0.7 mg/kg was calculated at 20 μM in the peripheral fluid. In the pulmonary tissue, the expression of iNOS was practically reversed after the injection of SsnB or MA (Figure 5B), thus presenting the anti-inflammatory activity of SsnB in vivo. The figures of the histological observation show that SsnB or MA significantly improved LPS-mediated pulmonary damage (Figure 5C,D).

## 3. Discussion 

This study demonstrated that SsnB was able to activate the expression of HO-1 dose-dependently. In addition, SsnB alleviated the LPS-stimulated levels of COX2/PGE2 and iNOS/NO and the NF-κB expression. NF-κB plays a major role in inflammatory processes, such as cellular proliferation, adjustment of cell differentiation, signaling of cell development, adhesive interplay, and anti-apoptosis activity [20]. Moreover, during systemic inflammatory responses, NF-κB regulates many immune activities through the facilitation of proinflammatory cytokines. Upregulated NO mediates inflammatory activities in the airway via controlling the expressions of chemokines, whereas LPS-induced production of iNOS and COX-2 needs sufficient activation of NF-κB [21]. Data obtained in this study showed that the SsnB-induced suppression of the synthesis of HO-1 and expressions of proinflammatory factors (iNOS, COX2, IL1-β and NO) were regulated via intervention of NF-κB activity. Furthermore, SsnB-induced suppression of the expression of iNOS and TNF-α in the BALF of LPS-treated mice might have been caused by HO-1. Based on the data of the present and prior works, we propose that SsnB influences inflammation-downregulating activity by modulating the HO-1 level, which represses oxidase response and/or NF-kB activation and lowers the levels of substrates derived by the phosphorylation of STAT-1. Moreover, SsnB enhanced the nuclear transmigration of Nrf2 and improved the ARE luciferase reporter activity. The SsnB-mediated regulation of iNOS production was controlled by the increase of HO-1, which implies that SsnB increased HO-1 production via inhibition of iNOS expression. 

These results are additionally assisted by the fact that the certain RNAi-mediated intervene inhibition of HO-1 remarkably recovered the suppression of SsnB in both NO and iNOS synthesis. In conclusion, this research shows that SsnB has a high effect in promoting the level of HO-1 and disturbs the synthesis of proinflammatory cytokines in LPS-administrated HUVECs; moreover, it decreased the expression of iNOS and TNF-α in lung tissues obtained from the LPS-treated mice. Overall, the presented results indicate the significance of HO-1 in breaking the inflammatory responses and prove that TNF-α is a mediator of the HO-1 pathway. For this reason, SsnB could be acknowledged as a promising perturbance for the clinical approach of inflammatory pathology, especially those related to the respiratory system.

## 4. Materials and Methods 

### 4.1. Cell Culture and Reagents

HUVECs were purchased from Cambrex BioScience (Charles City, IA, USA) and managed by the same protocol as the precedent research 11. SsnB, MA, LPS (L2654; isolated from *Escherichia coli*), penicillin G, streptomycin, and DMSO were commercially obtained from Sigma Chemical Co. (St. Louis, MO, USA). Human HO-1 (sc-35554) or control siRNA (sc-37007) were manufactured by Santa Cruz Biotechnology (Santa Cruz, CA, USA). Between 3 to 5 passages of subcultured HUVECs were acquired and seeded in a dish (density, 1 × 10^5^ cells per 35 mm diameter), making it nutrient-deficient overnight for ELISA. Some cells were conditioned with LPS (1 μg/mL for 6 h) and applied with SsnB for 6 h, and other cells were applied with SsnB for 6 h not treating LPS (to detect the level of HO-1).

### 4.2. Lung Injury Model by LPS Injection

Male C57BL/6 mice, 6 to 7 weeks old (average weight, 27 g), were purchased from Orient Bio Inc. (Seongnam, Korea) and before use for each experiment, were acclimatized for 12 days and were provided for adjustment as delineated [22,23]. Shortly, LPS (15 mg/kg i.p.) with 0.2% DMSO was administered with 28-gauge needles intraperitoneally. At 6 h after administration, intravenous (i.v.) injection of SsnB (0.04–0.4 mg/kg) was performed. The method was authorized by the Animal Care Committee at Kyungpook National University (IRB No. KNU 2017-102). BALF was obtained by following moderate suction after a phosphate-buffered saline (PBS) intra-tracheal administration, and centrifuge was conducted at 3000 rpm for 10 min at 4 °C. The final supernatant was kept in a freezer at −80 °C for following investigations.

### 4.3. ELISA

The HUVECs were stimulated by LPS (1 μg/mL, 6 h) and conditioned with SsnB for 6 h. SsnB was applied on the rest of the cells for 6 h without LPS activation to detect the concentration of HO-1. The levels of STAT1 phosphorylation were estimated by ELISA kits (Abcam, Cambridge, MA, USA). The upper fluid of the cell culture after the centrifuge were utilized to measure the levels of PGE2, HO-1, IL-1β, TNF-α, and iNOS. ELISA kits were manufactured by the R&D Systems (Minneapolis, MN, USA). 

### 4.4. Cell Viability Assay 

To verify the viability of cells, a 3-(4,5-dimethylthiazol-2-yl)-2,5-diphenyltetrazolium bromide (MTT) assay was performed, following the precedent research [22,23,24]. Living HUVECs in 96-well plates at a concentration of 5 × 10^3^ cells/well were cultured in an incubation with treatment of SsnB for 48 h. The cells were rinsed and stored in an incubator for another 4 h after applying 100 μL of MTT (1 mg/mL), respectively. At last, DMSO (150 μL) was added to dissolve the formed formazan salt. The level of formazan salt was measured (λ = 540 nm) with a spectrometer (Tecan, Austria GmbH, Grödig, Austria). 

### 4.5. Nitrite Levels

The production of nitric oxide was determined by estimating the concentration of nitrite (NO_2_^−^) in the cell media. The supernatant consisted of an identical quantity of Griess reagent (Abcam) and stored in an incubator for 15 min at room temperature. The activities were assessed on a spectrometer (λ = 540 nm). All evaluation was conducted with 3 trials.

### 4.6. Intracullular Fractionation and Immunoblotting

The cells were acquired and the supernatant was degraded by centrifuge. The cytosolic/nuclear essences were prepared, with regard to a precedent method [25]. Antibodies against iNOS, COX2, lamin B, Nrf2, and β-actin (Santa Cruz) were utilized for immunoblotting. Lamin B and β-actin are loading controls of the cytosolic/nuclear derivates, each.

### 4.7. Quantitative Real-Time-Polymerase Chain Reaction (qRT-PCR)

RNA was refined by TRI Reagent (Invitrogen, Waltham, MA, USA) extraction. The refined RNA was reverse-transcribed with a PX2 Thermal Cycler (Thermo Scientific, Waltham, MA, USA) with 0.5 mg/µL of the oligo (dT)-adapter primer (Invitrogen) and M-MLV reverse transcriptase (Invitrogen) in a 20 µL reaction compound. The expressions of iNOS and COX-2 were compared to β-actin. The following sequences of primers were designed and u for quantitative real-time-polymerase chain reaction (qRT-PCR) analysis: COX-2 forward: 5′-CCC CAT TAG CAG CCA GTT-3′, COX-2 reverse: 5′-CAT TCC CCA CGG TTT TGA-3′; iNOS forward: 5′-GTT CTC AGC CCA ACA ATA CAA GA-3′, iNOS reverse: 5′-GTG GAC GGG TCG ATG TCA C-3′; and β-actin forward: 5′-TCGTGCGTGACATCAAAGA-3′, β-actin reverse: 5′-CAT ACC CAA GAA GGA AGG CT-3′.

### 4.8. Plasmid Transfection

The preparation of NF-κB-luciferase reporter vector, HO-1 siRNA, ARE luciferase reporter vector, non-transcript control siRNA were processed by SuperFect (Qiagen, Germantown, MD, USA) and these prepared materials were utilized for plasmid transfections. After 4 h of transfection, the medium containing plasmid-transfected cells was replaced to novel medium.

### 4.9. ARE Luciferase Reporter Assay

The cells were purified with PBS of a mild temperature and disassembled by lysis buffer from the dual luciferase kit (Promega, Madison, WI, USA). The luciferase response was assessed using a TD-20/20 luminometer (Turner Designs, San Jose, CA, USA). All transfection procedures were performed in 3 independent trials. Results are illustrated as the percentage of the luciferase response of firefly to those of *Renilla*.

### 4.10. Histopathological Analysis

LPS is injected to the mice (*n* = 5) intraperitoneally (i.p.). After 6 h, they were treated with SsnB (0.58 mg/kg, i.v.), euthanized, and sacrificed. Hematoxylin and eosin (H&E) staining was done to investigate the histological variance among the lung tissue samples [24]. The pulmonary structure scores were estimated as grade 1 to grade 4, following the previous research [25].

### 4.11. Statistical Analysis

Figures are described as mean values ± SD of triple trials conducted respectively. One-way analysis of variance and Tukey’s post-hoc test were applied to demonstrate reasonable differences between each group. A *p* value of <0.05 was regarded to be statistically valid.

## References

[1] Chau L.Y. (2015). Heme oxygenase-1: Emerging target of cancer therapy. J. Biomed. Sci..

[2] Waza A.A., Hamid Z., Ali S., Bhat S.A., Bhat M.A. (2018). A review on heme oxygenase-1 induction: Is it a necessary evil. Inflamm. Res..

[3] Tsoyi K., Lee T.Y., Lee Y.S., Kim H.J., Seo H.G., Lee J.H., Chang K.C. (2009). Heme-oxygenase-1 induction and carbon monoxide-releasing molecule inhibit lipopolysaccharide (LPS)-induced high-mobility group box 1 release in vitro and improve survival of mice in LPS- and cecal ligation and puncture-induced sepsis model in vivo. Mol. Pharmacol..

[4] Ahmed S.M., Luo L., Namani A., Wang X.J., Tang X. (2017). Nrf2 signaling pathway: Pivotal roles in inflammation. Biochim Biophys Acta Mol. Basis Dis..

[5] Raghunath A., Sundarraj K., Nagarajan R., Arfuso F., Bian J., Kumar A.P., Sethi G., Perumal E. (2018). Antioxidant response elements: Discovery, classes, regulation and potential applications. Redox Biol..

[6] Liu H., Yu X., Yu S., Kou J. (2015). Molecular mechanisms in lipopolysaccharide-induced pulmonary endothelial barrier dysfunction. Int. Immunopharmacol..

[7] Liang Q.L., Wu Q.A., Jiang J.H., Duan J.A., Wang C., Smith M.D., Lu H., Wang Q., Nagarkatti P., Fan D.P. (2011). Characterization of Sparstolonin B, a Chinese Herb-derived Compound, as a Selective Toll-like Receptor Antagonist with Potent Anti-inflammatory Properties. J. Biol. Chem..

[8] Liang Q., Dong S., Lei L., Liu J., Zhang J., Li J., Duan J., Fan D. (2015). Protective effects of Sparstolonin B, a selective TLR2 and TLR4 antagonist, on mouse endotoxin shock. Cytokine.

[9] Zeng K.W., Zhang T., Fu H., Liu G.X., Wang X.M. (2012). Schisandrin B exerts anti-neuroinflammatory activity by inhibiting the Toll-like receptor 4-dependent MyD88/IKK/NF-kappaB signaling pathway in lipopolysaccharide-induced microglia. Eur. J. Pharmacol..

[10] Tang Y.M., Cao Q.Y., Guo X.Y., Dong S.H., Duan J.A., Wu Q.N., Liang Q.L. (2018). Inhibition of p38 and ERK1/2 pathways by Sparstolonin B suppresses inflammation-induced melanoma metastasis. Biomed. Pharmacother..

[11] Dattaroy D., Seth R.K., Das S., Alhasson F., Chandrashekaran V., Michelotti G., Fan D., Nagarkatti M., Nagarkatti P., Diehl A.M. (2016). Sparstolonin B attenuates early liver inflammation in experimental NASH by modulating TLR4 trafficking in lipid rafts via NADPH oxidase activation. Am. J. Physiol. Gastrointest Liver Physiol..

[12] Wang Y., Jiang S., Xiao J., Liang Q., Tang M. (2018). Sparstolonin B improves neurological outcomes following intracerebral hemorrhage in mice. Exp. Ther. Med..

[13] Wang M., Xiu L., Diao J., Wei L., Sun J. (2015). Sparstolonin B inhibits lipopolysaccharide-induced inflammation in 3T3-L1 adipocytes. Eur. J. Pharmacol..

[14] Liang Q., Yu F., Cui X., Duan J., Wu Q., Nagarkatti P., Fan D. (2013). Sparstolonin B suppresses lipopolysaccharide-induced inflammation in human umbilical vein endothelial cells. Arch. Pharm. Res..

[15] Lee W., Kim J., Park E.K., Bae J.S. (2020). Maslinic Acid Ameliorates Inflammation via the Downregulation of NF-kappaB and STAT-1. Antioxidants.

[16] Tsoyi K., Kim H.J., Shin J.S., Kim D.H., Cho H.J., Lee S.S., Ahn S.K., Yun-Choi H.S., Lee J.H., Seo H.G. (2008). HO-1 and JAK-2/STAT-1 signals are involved in preferential inhibition of iNOS over COX-2 gene expression by newly synthesized tetrahydroisoquinoline alkaloid, CKD712, in cells activated with lipopolysacchride. Cell. Signal..

[17] Tsoyi K., Nizamutdinova I.T., Jang H.J., Mun L., Kim H.J., Seo H.G., Lee J.H., Chang K.C. (2010). Carbon monoxide from CORM-2 reduces HMGB1 release through regulation of IFN-beta/JAK2/STAT-1/INOS/NO signaling but not COX-2 in TLR-activated macrophages. Shock.

[18] Lee W., Lee D., Lee Y., Lee T., Song K.S., Yang E.J., Bae J.S. (2018). Isolation, Synthesis, and Antisepsis Effects of a C-Methylcoumarinochromone Isolated from Abronia nana Cell Culture. J. Nat. Prod..

[19] Lee W., Park S.Y., Yoo Y., Kim S.Y., Kim J.E., Kim S.W., Seo Y.K., Park E.K., Kim I.S., Bae J.S. (2018). Macrophagic Stabilin-1 Restored Disruption of Vascular Integrity Caused by Sepsis. Thromb. Haemost..

[20] Wullaert A., Bonnet M.C., Pasparakis M. (2011). NF-kappaB in the regulation of epithelial homeostasis and inflammation. Cell Res..

[21] Lee W., Ahn J.H., Park H.H., Kim H.N., Kim H., Yoo Y., Shin H., Hong K.S., Jang J.G., Park C.G. (2020). COVID-19-activated SREBP2 disturbs cholesterol biosynthesis and leads to cytokine storm. Signal Transduct Target Ther..

[22] Jeong S.Y., Kim M., Park E.K., Kim J.-S., Hahn D., Bae J.-S. (2020). Inhibitory Functions of Novel Compounds from Dioscorea batatas Decne Peel on HMGB1-mediated Septic Responses. Biotechnol. Bioprocess Eng..

[23] Lee I.-C., Ryu C.-W., Bae J.-S. (2020). Novel Herbal Medicine C-KOK Suppresses the Inflammatory Gene iNOS via the Inhibition of p-STAT-1 and NF-κB. Biotechnol. Bioprocess Eng..

[24] Lee W., Ku S.K., Kim J.E., Cho G.E., Song G.Y., Bae J.S. (2019). Pulmonary protective functions of rare ginsenoside Rg4 on particulate matter-induced inflammatory responses. Biotechnol. Bioprocess Eng..

[25] Kim J.E., Lee W., Yang S., Cho S.H., Baek M.C., Song G.Y., Bae J.S. (2019). Suppressive effects of rare ginsenosides, Rk1 and Rg5, on HMGB1-mediated septic responses. Food Chem. Toxicol..

